# Synthesis and Evaluation of New Fluorinated *Anti*-*Tubercular* Compounds

**Published:** 2014

**Authors:** Marjan Esfahanizadeh, Koroush Omidi, Joel Kauffman, Ali Gudarzi, Shahram Shahraki Zahedani, Salimeh Amidi, Farzad Kobarfard

**Affiliations:** a *Department of Medicinal Chemistry, School of Pharmacy, Shahid Beheshti University of Medical Sciences, Tehran, Iran. *; b *Central Research Laboratories, Shahid Beheshti University of Medical Sciences, Tehran, Iran. *; c *Department of Chemistry and Biochemistry, University of the Sciences in Philadelphia, Philadelphia, PA, USA. *; d *Department of Microbiology, School of Medicine, Zahedan University of Medical Sciences, Zahedan, Iran. *; e *Phytochemistry Research Center, Shahid Beheshti University of Medical Sciences, Tehran, Iran.*

**Keywords:** Fluorinated analogs, Anti-*tuberculosis* drugs, Pyrazinamide, PAS, Thiacetazone

## Abstract

Treatment of *tuberculosis* (TB) and the discovery of effective new anti-*tubercular* drugs are among the most urgent priorities in health organizations all over the world. In the present study, fluorinated analogs of some of the most important anti-TB agents such as p-aminosalicylic acid (PAS), thiacetazone and pyrazinamide were synthesized and tested against TB. The fluorinated analog of thiacetazone was 20 times more potent than the parent compound against *M.tuberculosis* H_37_-R_V_, while the fluorinated p-aminosalicylic acid (PAS) was almost three times less potent than PAS. A few other halogenated analogs of thioacetazone were also synthesized and subjected to anti-*M.tuberculosis* screening tests. The best halogen substituent was found to be fluorine which has the smallest size from one hand and the strongest electronegativity from the other hand among the halogen atoms. Fluorine therefore could be considered as a golden substituent to improve the anti-*M.tuberculosis* activity of thioacetazone.

## Introduction

*Tuberculosis* is the oldest documented infectious disease and it remains an important health concern all around the world. As indicated by recent reports of WHO, there were an estimated 8.7 million new cases of TB (13% co-infected with HIV) in 2011 and 1.4 million people died from TB in this year (1). The global resurgence of *tuberculosis* and development of drug resistance have rekindled the need for the development of new anti-*tubercular* drugs (2, 3). The results have been a torrent of papers and reports on compounds, which exhibit new mechanism of action or improved efficacy. Fluorine has been an attractive group for medicinal chemists and fluorinated compounds have many advantages that some of them are (4): a. as the second smallest substituent, fluorine most closely mimics hydrogen with respect to steric requirements at enzyme receptor sites, b. the presence of fluorine often leads to increased lipid solubility, thereby enhancing rates of absorption and transport of drugs *in-vivo*, c. the high electronegativity of fluorine frequently alters electronic properties, and thereby chemical reactivity and physical properties of compounds, d. fluorine imparts increased oxidative and thermal stability because the C-F bond is stronger than the C-H bond. This also limits enzymatic deactivation, e. in special cases, *e.g*. 5-fluorouracil, the specific location of the "deceptor" fluorine instead of hydrogen blocks an essential biochemical reaction and leads to its tumor inhibitory behavior. 

Fluorinated analog of isoniazide as, one of the most famous drugs for *tuberculosis* treatment, has already been synthesized and the results show that introducing fluorine on the pyridine ring drastically decreases the activity against *M. tuberculosis *(5)*.* However the fluorinated derivatives of other first line medications in *tuberculosis* treatment *i.e*. PAS, pyrazinamide and thioacetazone have not been reported yet.

**Figure 1:**
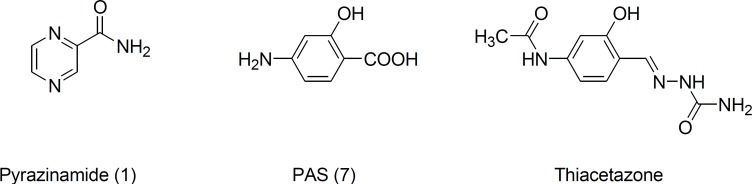


As part of our efforts to find new anti-TB agents, we have synthesized a series of fluorinated derivatives of famous synthetic anti-*tuberculosis* agents such as pyrazinamide, p-aminosalicylic acid (PAS) and thiacetazone and their activities against *M. tuberculosis *were evaluated (Figure 1).

*Chemistry*

**Figure 2:**
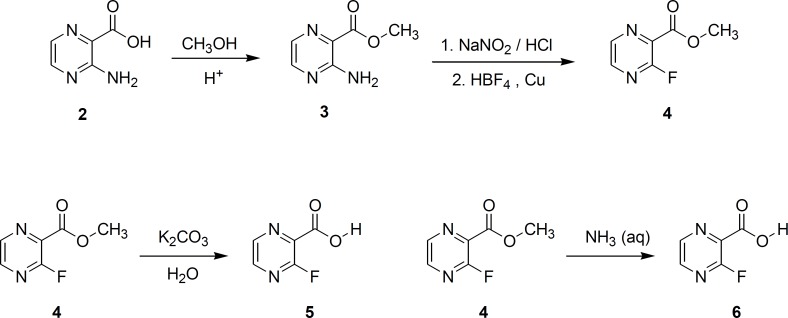
Preparation of flourinated analogs of pyrazinamide

Substitution of fluorine on aromatic ring is conventionally performed via diazotization of primary aromatic amines, followed by thermal decomposition of the corresponding diazonium perfluoroborate salt (Schiemann reaction). This method was used for the preparation of 3-fluoropyrazincarboxamide (compound 6) and the desired compound was obtained in satisfactory yield (Figure 2).

In case of PAS and thioacetazone, the aromatic ring is rather electron rich and direct fluorination via an electrophilic aromatic substitution could be considered as the plausible method for the synthesis of their fluorinated derivatives. 

**Figure 3:**
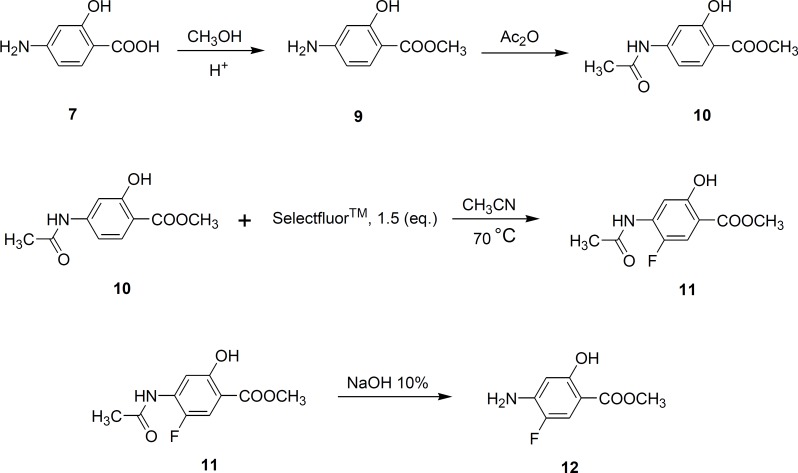
Synthesis of Fluorinated derivative of PAS

The carboxylic acid group in PAS was first converted to its methyl ester form, followed by acetylation of 4-amino group in acetic anhydride. Selectfluor^TM^ (6) (1-chloromethyl-4-fluoro-1,4-diazoniabicyclo [2.2.2] octane bistetrafluoroborate) was used (1.5 equivalent) for direct fluorination of the aromatic ring at position 5 to obtain compound 11. Hydrolysis of this compound in 10% sodium hydroxide solution gave 4-amino-5-fluorosalicylic acid (compound 12) (Figure 3).

**Figure 4:**
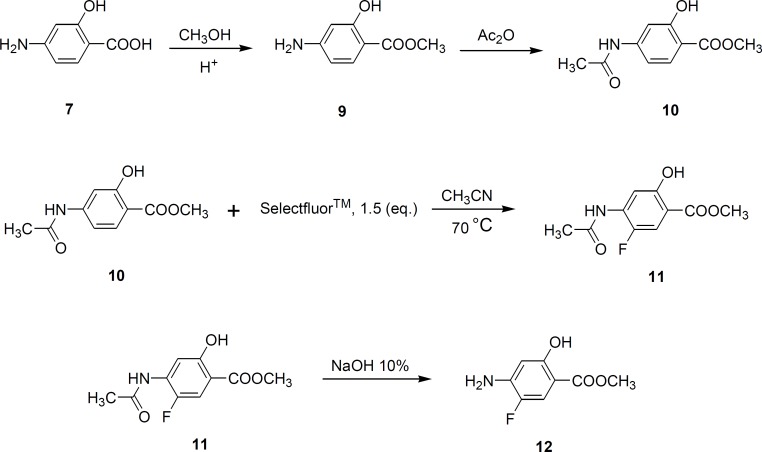
Synthesis of fluorinated analog of thiacetazone

3-fluoro-4-acetamidobenzaldehyde thiosemicarbazone (compound 16) was synthesized according to the synthesis scheme depicted in Figure 4. 4-acetamidobenzaldehyde was subjected to direct fluorination by Selectfluor^TM^ in acetonitrile. 3-Fluoro-4-acetamidobenzaldehyde thus obtained, was reacted with thiosemicarbazide to obtain compound 16 in satisfactory yield (Figure 4).

**Figure 5:**
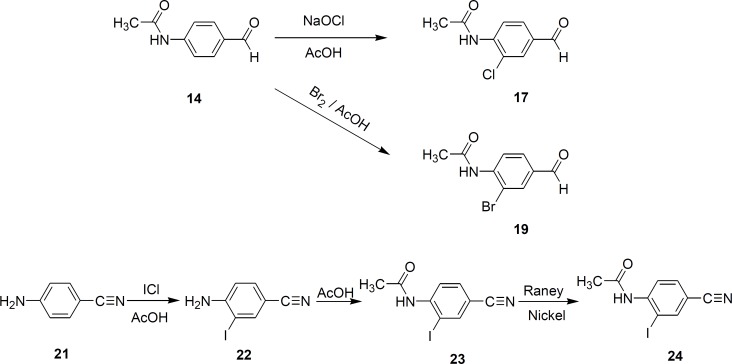
synthesis of halogenated derivatives of thiacetazone

In order to investigate the role of fluorine substitution on the activity of thiacetazone, other halogenated derivatives of this compound were also synthesized and subjected to anti-*mycobacterium* *tuberculosis* screening tests.

Figure 5 illustrates the synthetic routes for the synthesis of compounds 17-25 (7, 8). Halogenated derivatives of PAS and pyrazinamide had been reported previously in literature as less active and therefore no attempt was made for their synthesis and anti-*tuberculosis* tests (9).

## Experimental

*Chemistry*

All solvents, reagents and catalysts were of analytical grade and used without further purification. The melting points (˚C) were determined by open capillary method on an Electrothermal melting point apparatus and were uncorrected. The purity of compounds was confirmed by thin layer chromatography using Whatman Sil G/UV_254_ silica gel plates as the stationary phase and with suitable mobile phase and the spots were visualized under 254 and 366 nm illumination. Infrared spectra were recorded as thin films on KBr plates with υ_max_ in inverse centimeters. ^1^H NMR spectra were recorded on a Bruker DRX-Avance (500 MHz) and or (250 MHz) spectrometer using DMSO-d6 and CDCl_3_ as solvents and chemical shift values are expressed in ppm (parts per million) relative to tetramethylsilane (TMS) as internal standard; s = singlet, d = doublet, dd = doublet of doublets, t = triplet, q = quartet, m = multiplet, brs = broad singlet. Mass analyses were performed on an Agilent 6400 Series equipped with an electrospray ionization source (capillary voltage at 4000V, nebulizing gas temperature at 300 ˚C, nebulizing gas flow at 12 L/min). All the compounds were analyzed for C, H, N and S on a Costech model 4010 and agreed with the proposed structures within ± 0.4% of the theoretical values.

*Preparation of methyl 3-aminopyrazine-2-carboxylate (3)*

To a suspension of 0.06 mol 3-aminopyrazine-2-carboxylic acid (2) in 40 ml methanol was added 8 mL of concentrated sulfuric acid slowly. The mixture was heated under reflux at 65 ˚C for 1.5 hours and was allowed to cool to room temperature. Enough concentrated ammonium hydroxide solution was added to make the pH basic and the precipitate was filtered and dried, resulting in 4.68 g (51%) of 3 as yellow solid. mp 169-171.5 ˚C. IR (KBr): 3446, 3257, 3153, 1696 (C=O), 1610, 1121 cm^-1^; ^1^H NMR (90 MHz, DMSO-d6) δ 8.3 (^1^H; d, J_H5-H6_ = 2.2Hz; H-6), 7.94(^1^H; d, J_H6-H5_ = 2.2Hz; H-5), 7.36 (2H, brs, NH_2_), 3.88 (3H, s, CH_3_)

*Preparation of methyl 3-fluoropyrazine-2-carboxylate (4)*

A mixture of 0.0215 mole methyl 3-aminopyrazine-2-carboxylate (3) and 0.05 g copper powder in 12.5 mL of 40% tetrafluoroboric acid was stirred at room temperature for 10 minutes. After cooling to -5 ˚C in an ice-acetone bath, 1.79 g (0.026 mol) sodium nitrite was added in small portions while maintaining the temperature below 5 ˚C. The suspension was stirred at 0 ˚C for 15 minutes followed by 1.5 hours at room temperature. The reaction mixture was neutralized to a pH of 5-6 with sodium carbonate at 5-10 ˚C and extracted several times with 100 mL portions of diethyl ether. The combined ether layers were dried over anhydrous magnesium sulfate and the solvent was evaporated to give 2.75 g of the crude product. This compound was subjected to sublimation at 90 ˚C, yielding 1.4 g (22%) of 4 as white crystals, mp: 44-46 ˚C. IR (thin film of NaCl plate): 1740 (C=O), 1413, 1277, 1109 cm^-1^. ^ 1^H NMR (90 MHz, DMSO-d6): δ 8.82 (^1^H; dd, J_F-H5_ = 4.2 Hz, J_H6-H5 _= 2.4 Hz; H-5), 8.67 (^1^H; dd, J_H5-H6_ = 2.4 Hz, J_F-H6_ = 1.6 Hz; H-6), 3.97 (3H, s, CH_3_).

*Preparation of 3-fluoropyrazine-2-carboxylic Acid (5)*

To a solution of 0.3 g (0.0022 mol) potassium carbonate in 7 mL water, was added 0.624 g (0.004 mol) methyl 3-fluoropyrazine-2-carboxylate (4). The mixture was heated at 90 ˚C under reflux for one hour and then was allowed to cool to room temperature. Enough concentrated HCl was added to bring the pH to 1. The precipitate was filtered and dried to give 0.34 g (60%) of a white solid. Recrystallization of this compound from water gave 0.27 g (42%) of the pure product which was characterized as 3-fluoro-2-pyrazinecarboxylic acid monohydrate, mp 106-108.5˚C. IR (potassium bromide): 3527, 1716 (C=O), 1585, 1410, 1117 cm^-1^. ^1^H NMR (90 MHz, DMSO-d6): δ 8.8 (^1^H; dd, J_F-H5_ = 4.2 Hz, J_H6-H5 _= 2.5 Hz; H-5), 8.62 (^1^H; dd, J_H5-H6_ = 2.5 Hz, J_F-H6_ = 1.6 Hz; H-6), 6.8 (^1^H, brs, COOH + H_2_O). 

*Anal. *Calcd. for C_5_H_3_FN_2_O_2_.H_2_O (160.10): C, 37.5; H, 3.15; F, 11.57; N, 17.5. Found: C, 37.33; H, 2.68; F, 11.8; N, 17.62.

*Preparation of 3-fluoropyrazine-2-carboxamide (6)*

A mixture of 0.38 g (0.0024 mol) methyl 3-fluoropyrazine-2-carboxylate (4) in 5 mL concentrated ammonium hydroxide solution was stirred at room temperature for 10 minutes. The precipitate was filtered and recrystallized from water to give 0.134 g (40%) of 6 as white crystals. mp: 170-172 ˚C. IR (potassium bromide): 3438, 3155, 1710 (C=O), 1578, 1381 cm^-1^. ^1^H NMR (90). MHz, DMSO-d6): δ 8.7 (^1^H; dd, J_F-H5_ = 4.2 Hz, J_H6-H5 _= 2.5 Hz; H-5), 8.55 (^1^H; dd, J_H5-H6_ = 2.5 Hz, J_F-H6_ = 1.6 Hz; H-6), 8 (2H, br d, NH_2_). *Anal. *Calcd. for C_5_H_4_FN_3_O (141.10): C, 42.56; H, 2.56; F, 13.46; N, 29.78. Found: C, 42.82; H, 3.19; F, 13.10; N, 30.13.

*Preparation of methyl 4-aminosalicylate (9)*

To a suspension of 9.18 g (0.06 mol) 4-aminosalicylic acid in 40 mL of dry methanol was added 8 mL of concentrated sulfuric acid slowly. The mixture was heated under reflux at 70 ˚C for 1.5 hours and then it was cooled in an ice-water bath. Enough concentrated ammonium hydroxide solution was added to adjust the pH to 9 and the precipitate was filtered, rinsed with water and dried to give 6.01 g (60%) of 9 as a solid, mp 118-120 ˚C. IR (potassium bromide): 3473 and 3379 (NH_2_), 1643 (C=O), 1284 cm^-1^. ^1^H NMR (90 MHz, CDCl_3_): δ 10.96 (1H, s, OH), 7.6 (^1^H; d, J_H5-H6_ = 9 Hz; H-6), 6.2-6.08 (2H, cm, H-3 and H-5), 4.2 (2H; brs; NH_2_), 3.87 (3H, s, CH_3_).

*Preparation of methyl 4-acetamidosalicylate (10)*

To a suspension of 4.17 g (0.025 mol) methyl 4-aminosalicylate (9) in 20 mL water, was added 3 mL (0.032 mol) acetic anhydride while stirring. The mixture was heated at 80 ˚C for 30 minutes and cooled to room temperature. The precipitate was collected and added into 100 mL of 10% hydrochloric acid. This suspension was stirred at room temperature for 10 minutes, filtered and dried and recrystallized from methanol yielding 3 g (70%) of 10. mp: 153-154 IR (potassium bromide): 3319 (NH), 1680 (C=O), 1604, 1157 cm^-1^.^ 1^H NMR (90 MHz, CDCl_3_ + DMSO-d6): δ 10.8 (^1^H, s, OH), 9.74 (^1^H; brs; NH), 7.73 (^1^H; d, J_H5-H6_ = 9 Hz; H-6), 7.37 (^1^H; d, J_H5-H3_ = 1.8 Hz; H-3), 7.11 (^1^H; dd, J_H6-H5_ = 9 Hz; J_H3-H5_ = 1.8 Hz, H-5), 3.91 (3H, s, OCH_3_), 2.15 (3H, s, CH_3_).

*Preparation of methyl 4-acetamido-5-fluorosalicylate (11)*

A solution of 10.62 g (0.03 mol) Selectfluor^TM^ in 200 mL acetonitrile was obtained by heating the mixture at 70-80 ˚C. Then 4.18g (0.2 mol) methyl 4-acetamidosalicylate (10) was added and the solution was heated under reflux for 4.5 hours at 80 ˚C. The reaction mixture was allowed to cool down and added into 350 mL of diethyl ether. The mixture was washed first with 4 × 250 ml water and then with 150 mL saturated solution of sodium bicarbonate, dried over anhydrous magnesium sulfate, evaporated, and recrystallized from methanol twice to give 1 g (22%) of 11. mp: 169-172.5

IR (potassium bromide): 3294 (NH), 1681 (C=O), 1630 (C=O), 1547, 1260, 1185 cm^-1^. ^1^H NMR (90 MHz, CDCl_3_ + DMSO-d6): δ 10.56 (^1^H, s, OH), 9.86 (^1^H; brs; NH), 7.96 (^1^H; d, J_F-H3_ = 7.2Hz; H-3), 7.49 (^1^H; d, J_F-H6_ = 11.7 Hz; H-6), 3.93 (3H, s, OCH_3_), 2.22 (3H, s, CH_3_).* Anal. *Calcd. for C_10_H_10_FNO_4_ (227.19): C, 52.87; H, 4.44; F, 8.36; N, 6.17. Found: C, 52.86; H, 4.43; F, 7.89; N, 6.17. 

*Preparation of 4-amino-5-fluorosalicylic acid (12)*

A solution of 1 g (0.0047 mol) methyl 4-acetamido-5-fluorosalicylate (11) in 20 mL of 20% sodium hydroxide solution was heated under reflux for 2 hours and was cooled. Enough concentrated HCl was added to bring the pH to 2. The precipitate was filtered, dried and recrystallized from water/methanol, giving 12 as white crystals. mp: 171-172 ˚C.

IR (potassium bromide): 3486 & 3380 (NH_2_), 1656 (C=O), 1535, 1446 cm^-1^. ^1^H NMR (90 MHz, Acetone-d_6_): δ 10.99 (2H, very b s, OH and COOH), 7.4 (^1^H; d, J_F-H6_= 11.7 Hz; H-6), 6.33 (1H; d, J_F-H3_= 7.2 Hz; H-3), 5.74 (2H, br s, NH_2_). *Anal. *Calcd. for C_7_H_6_FNO_3 _(171.12): C, 49.13; H, 3.53; F, 11.10; N, 8.18. Found: C, 48.91; H, 3.62; F, 11.12; N, 8.03.

*Preparation of 4-acetamido-3-fluorobenzaldehyde (15)*

A solution of 20.56 g (0.058 mol) Selectfluor^TM^ in 400 mL acetonitrile was obtained by heating the mixture at 70-80 ˚C. To this solution was added 4.73 g (0.029 mol) 4-acetamidobenzaldehyde and the mixture was heated at 70 ˚C under reflux for 72 hours. The reaction mixture was allowed to cool and then added into 500 mL diethyl ether. The mixture was washed first with 3 × 300 mL water and then with 300 mL of saturated solution of sodium bicarbonate, dried over anhydrous MgSO_4_, evaporated and recrystallized from water containing 1 g activated charcoal to yield 0.91 g (19%) of 15. mp: 131-133 ˚C (ref. 133-135 ˚C)

IR (potassium bromide): 3256 (NH), 1674 (C=O), 1609, 1535, 1431, 1257 cm^-1^. ^1^H NMR (90 

MHz, DMSO-d6): δ 10.1 (^1^H, br s, NH), 9.9 (^1^H; d, J = 2Hz; formyl H), 8.36 ( ^1^H; t, J = 8Hz; H-5), 7.81-7.7 (2H; m, H-2 and H-6), 2.17 (3H, s, CH_3_).

*Preparation of 4-acetamido-3-fluorobenzaldehyde thiosemicarbazone (16)*

A solution of 0.18 g (0.002 mol) thiosemicarbazide in 6 mL water containing 0.4 mL acetic acid was added to a solution of 0.36 g (0.002 mol) 4-acetamido-3-fluorobenzaldehyde (15) in 5 mL ethanol at 70 ˚C. The mixture was stirred at this temperature for 30 minutes. A white precipitate developed in the solution, which was filtered after cooling the reaction mixture. This compound was recrystallized from ethanol twice, yielding 0.17 g of 16. mp: 231 ˚C.

IR (potassium bromide): 3358, 3293, 3167, 1666 (C=O), 1581, 1286 cm^-1^. ^1^H NMR (90 

MHz, DMSO-d6): δ 11.49 (^1^H; s, thiosemicarbazone NH), 9.86 (^1^H, s, amide NH), 8.22-7.4 (5H, m, aromatic Hs and NH_2_), 2.13 (3H, s, CH_3_).* Anal. *Calcd. for C_10_H_11_FN_4_OS (254.28): C, 47.23; H, 4.36; F, 7.47; N, 22.03; S, 12.61. Found: C, 47.25; H, 4.53; F, 6.89; N, 22.07; S, 12.30.

*Preparation of 4-acetamido-3-chlorobenzaldehyde (17)*

To a solution of 6.43 g (0.039 mol) 4-acetamidobenzaldehyde in 55 mL of glacial acetic acid, was added 100 mL of 5.25% solution of sodium hypochlorite and the reaction mixture was stirred at room temperature for 48 hours. The mixture was poured into 100 mL water and filtered to give 2.5 g (32%) of 17. mp: 110-113 ˚C.

IR (potassium bromide): 3334 (NH), 1706 (C=O), 1688 (C=O), 1575, 1527, 1375 cm^-1^. ^1^H NMR (90 MHz, DMSO-d6): δ 9.95 (^1^H, s, formyl H), 9.74 (^1^H, br s, NH), 8.21 (^1^H; d, J_H6-H5 _= 9 Hz; H-5), 8.02 (^1^H; d, J_H6-H2 _= 1.8 Hz; H-2), 7.87 (^1^H; dd, J_H5-H6_ = 9.0 Hz, J_H2-H6_ = 1.8 Hz; H-6), 2.22 (3H, s, CH_3_).

*Preparation of 4-acetamido-3-chlorobenzaldehyde thiosemicarbazone (18)*

A solution of 0.73 g (0.008 mol) of thiosemicarbazide in 24 mL of water containing 1.6 mL glacial acetic acid was added to a solution of 1.58 g of (0.008 mol) 4-acetamido-3-chlorobenzaldehyde (17) in 20 mL of ethanol at 70 ˚C. The mixture was stirred at this temperature for 45 minutes. A white precipitate developed in the reaction mixture, which was filtered after cooling to give 1.95 g (90%) of 18. mp: 235-238 ˚C.

IR (potassium bromide): 3423, 3260, 3132, 1701 (C=O), 1594, 1508, 1303 cm^-1^. ^1^H NMR (90 MHz, DMSO-d6): δ 11.47 (^1^H, s, thiosemicarbazone NH), 9.35 (^1^H, s, amide NH), 8.15-7.91 (5H; m; H-2, H-5, imine H and thiosemicarbazone NH_2_), 7.6 (^1^H; dd, J_H5-H6_ = 8.1 Hz, J_H2-H6_ = 1.8 Hz; H-6), 2.18 (3H, s, CH_3_).

*Anal. *Calcd. for C_10_H_11_ClN_4_OS (270.73): C, 44.36; H, 4.10; Cl, 13.10; N, 20.69; S, 11.84. Found: C, 44.51; H, 4.14; Cl, 12.90; N, 20.67; S, 12.27.

*Preparation of 4-acetamido-3-bromobenzaldehyde thiosemicarbazone (20)*

A solution of 1.32 mL (0.0265 mol) of bromine in 6.25 mL of glacial acetic acid was added to a solution of 4.07 g (0.025 mol) of 4-acetamidobenzaldehyde in 22 mL glacial acetic acid slowly at room temperature. A precipitate developed in the reaction mixture when almost half of the bromine solution was added. The mixture was stirred at room temperature for one hour further and then poured into 100 mL of water. The mixture was stirred for 30 minutes until the strong yellow color of the solution disapeared. The precipitate was filtered and dried. Several recrystallization from methanol did not yield a pure compound. To a solution of 0.48 g of this mixture in 5 mL of ethanol, was added a solution of 0.182 g of thiosemicarbazide in 6 mL of water containing 0.4 mL of acetic acid at 70 ˚C. The mixture was stirred at this temperature for 45 minutes. The precipitate was filtered without cooling the mixture to give 0.2 g of 20. mp: 232-235 ˚C. 

IR (potassium bromide): 3418, 3235, 3146, 1690 (C=O), 1598, 1520, 1299 cm^-1^. ^1^H NMR (90 MHz, DMSO-d6): δ 11.5 (^1^H, s, thiosemicarbazone NH), 9.47 (^1^H, s, amide NH), 8.25-7.72 (6H; m; aromatic Hs, imine H and thiosemicarbazone NH_2_), 2.12 (3H, s, CH_3_).

*Anal. *Calcd. for C_10_H_11_BrN_4_OS (315.18): C, 38.11; H, 3.50; Br, 25.35; N, 17.78; S, 10.17. Found: C, 38.58; H, 3.74; Br, 24.98; N, 17.94; S, 11.52.

*Preparation of 4-amino-3-iodobenzonitrile (22)*

To a solution of 5.9 g (0.05 mol) of 4-aminobenzonitrile in 25 mL of glacial acetic acid was added dropwise a solution of 0.05 mol of iodine monochloride in 5 mL of glacial acetic acid. During the addition the temperature rose to 40 ˚C. The solution was stirred at room temperature for 20 minutes. A solid developed in the reaction mixture and the deep brown color of the solution started fading gradually. The mixture was poured into 250 mL of water and stirred for 10 minutes to give a solid which was filtered and recrystallized from methanol/water containing 1 g of activated charcoal yielding 9.3 g (76%) of 22. mp: 110-112 ˚C.

IR (potassium bromide): 3454 and 3346 (NH_2_), 2214 (CN), 1621, 1496 cm^-1^. ^1^H NMR (90 MHz, CDCl_3_): δ 7.91 (^1^H; d, J_H6-H2_ = 1.8 Hz; H-2), 7.41 (^1^H; dd, J_H5-H6_ = 8.4 Hz, J_H2-H6_ = 1.8 Hz; H-6), 6.73 (1H; d, J_H6-H5 _= 8.4 Hz; H-5), 4.67 (2H, br s, NH_2_).

*Preparation of 4-acetamido-3-iodobenzonitrile (23)*

A mixture of 8.54 g (0.035 mol) of 4-amino-3-iodobenzonitrile (22), 16 mL (0.16 mol) of acetic anhydride and five drops of concentrated sulfuric acid was heated at 70 ˚C under reflux for 10 minutes. The reaction mixture was poured over 400 mL of cold water and stirred for 5 minutes to give a white solid which was filtered and dried, yielding 9.48 g (95%) of 23. mp: 176-181 ˚C. IR (potassium bromide): 3276 (NH), 1663 (C=O), 2230 (CN), 1517, 1297 cm^-1^. ^1^H NMR (90 MHz, CDCl_3_): δ 8.73 (^1^H, br s, NH), 8.13-8.05 (2H, m, H-5 and H-2), 7.65 (^1^H; dd, J_H5-H6_ = 8.4 Hz, J_H2-H6_ = 1.8 Hz; H-6), 2.25 (3H, s, CH_3_).

*Preparation of 4-acetamido-3-iodobenzaldehyde (24)*

A mixture of 0.0197 mol of 4-acetamido-3-iodobenzonitrile (23) 3.6 g of Raney nickel and 55 mL of 75% formic acid was heated under reflux at 85 ˚C for 1.5 hours. While the reaction mixture was still hot, it was filtered through a cake of filter aid and the residue was washed with 3 × 10 mL of absolute ethanol. The solvent was evaporated and the solid was recrystallized from methanol/water, yielding 4.2 g (73%) of 24. Mp: 145-147 ˚C.

 IR (potassium bromide): 3272 (NH), 1700 (C=O), 1565, 1524, 1368, 1198 cm^-1^. ^1^H NMR (90 MHz, CDCl_3_): δ 9.86 (^1^H, s, formyl H), 8.51 (^1^H; d, J_H6-H5_ = 8.6 Hz; H-5), 8.31 (^1^H; d, J_H6-H2_ = 1.8 Hz; H-2), 7.85 (1H; dd, J_H5-H6_ = 8.6 Hz, J_H2-H6_ = 1.8 Hz), 7.7 (1H, br s, NH), 2.3 (3H, s, CH_3_).

*Preparation of 4-acetamido-3-iodobenzaldehyde thiosemicarbazone (25)*

A solution of 0.008 mol of thiosemicarbazone in 25 mL of water containing 1.6 mL of glacial acetic acid was added to a solution of 0.008 mol 4-acetamido-3-iodobenzaldehyde (24) in 40 mL of absolute ethanol at 80 ˚C. The mixture was stirred at this temperature for 45 minutes. A white precipitate developed in the solution which was filtered after cooling the reaction mixture to give 2.55 g (88%) of 25. mp: 241-243 ˚C.

IR (potassium bromide): 3382, 3242, 3153, 1694 (C=O), 1592, 1502, 1296 cm^-1^. ^1^H NMR (90 MHz, DMSO-d6): δ 11.5 (^1^H, s, thiosemicarbazone NH), 9.42 (^1^H, s, amide NH), 8.43 (^1^H; d, J_H2-H6_ = 1.7 Hz; H-2), 8.2 (2H, br s, thiosemicarbazone NH), 8.0 (^1^H, s, imine H), 7.76 (^1^H; dd, J_H5-H6_ = 8.5 Hz, J_H2-H6_ = 1.7 Hz; H-6), 7.52 (^1^H; d, J_H6-H5_ = 8.5 Hz; H-5), 2.1 (3H, s, CH_3_).

*Anal. *Calcd for C_10_H_11_IN_4_OS (315.18): C, 33.16; H, 3.06; I, 35.04; N, 15.47; S, 8.85. Found: C, 33.29; H, 3.18; I, 35.09; N, 15.35; S, 9.3.

*Anti-tuberculosis activity:*

*In-vitro evaluation of anti-mycobacterial activity*

Primary screening was conducted at 6.25 µg/ mL of the tested compounds against *Mycobacterium tuberculosis* H_37_Rv in BACTEC 12B medium using a broth microdilution assay, the Microplate Alamar Blue Assay (MABA) (10). Compounds effecting < 90% inhibition in primary screen (*i.e*., MIC > 6.25 µg/mL) were not generally evaluated further. Compounds demonstrating at least 90% inhibition in primary screening were retested at lower concentration against *M.* *tuberculosis *H_37_R_V_ to determine the actual minimum inhibitory concentration (MIC) using MABA. The MIC is defined as the lowest concentration effecting a reduction in fluorescence of 90% relative to controls. 

Concurrent with the determination of MIC, compounds were tested for cytotoxicity (IC_50_) in VERO cells at concentration ≤ 62.5 µg/ mL or 10 × the MIC for *M*. *tuberculosis *H_37_R_V . _ After 72 hours exposure, viability was assessed on the basis of cellular conversion of MTT into a formazan product using the Promega Cell Titer 96 Non-radioactive Cell Proliferation Assay.

For the most active compounds MICs were determined in MABA against *M. avium* and against five strains of single-drug-resistant (SDR) *M. tuberculosis *(*i.e*., each strain resistant to a single TB drug). The minimum bactericidal concentration (MBC) was then determined for *M*. *tuberculosis *H_37_R_V _and Erdman (and for the appropriate drug-resistant strain, for analogs of known anti-*tubercular* drugs) by subculturing on drug-free solid media and enumeration colony forming units following exposure in supplemented Middlebrook 7H9 media to drug concentrations equivalent to and higher than the previously determined MICs of the respective strains.

## Results and Discussion

All the synthesized fluorinated compounds were assayed for their anti-*tubercular* activities and the results of primary screening are presented in Table 1.

The Bactec media, which is used for these tests, has the pH of 6.5-6.8. Pyrazinamide itself is almost completely inactive at a neutral pH (11). Thus one could expect to get almost no activity for the compounds 5 and 6 which are close derivatives of pyrazinamide.

**Table 1:** Anti-*Mycobacterium tuberculosis* activity of the fluorinated compounds

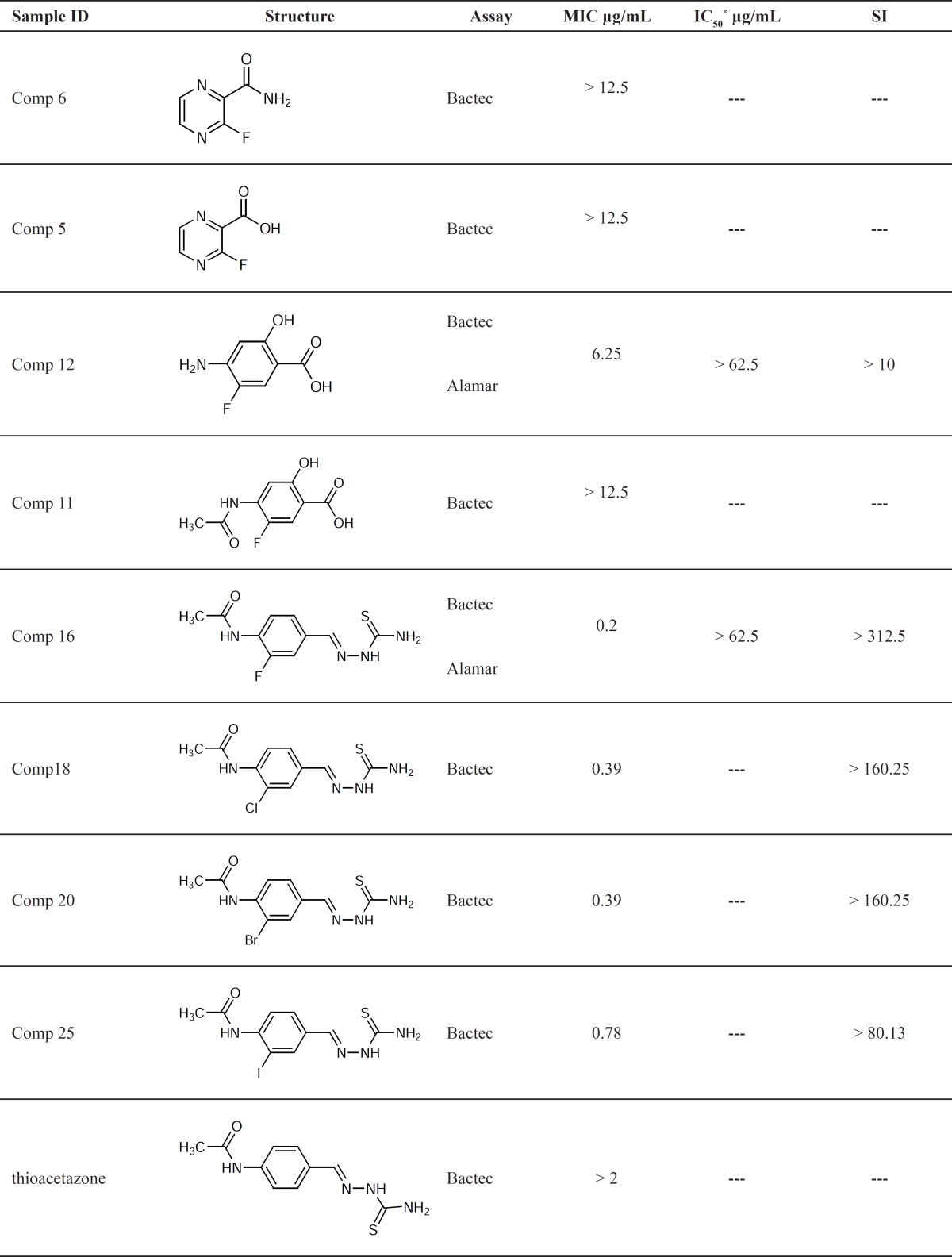

*IC_50_:Cytotoxicity in VERO cells

Comment: MIC of rifampicin: 0.015 µg/ mL

IC_50 _of rifampicin: 77.7

MIC of INH: 0.025 µg/ mL

IC_50_ of INH: 601.6

†SI: Selectivity Index (Calculated by dividing IC50 to MIC.(

MIC of *p-*aminosalicylic acid (PAS) against *M. tuberculosis *H_37_R_V _(determined by MABA system) is 1.25 µg/ mL (10), while this value for compound 12 is ≤ 3.13. This means compound 12 is almost three times less active than the parent compound; but it is still considered as a potential candidate and further tests were conducted on this compound and the results are presented in Table 2.

The MIC of thiacetazone against *M*. *tuberculosis *H_37_R_V _(determined by MABA system) is > 2.0 µg/mL (10), while this value for compound 16 is ≤ 0.1. This means compound 16 is about 20 times more potent than the parent compound; and it is an excellent candidate for further tests (Table 2).

Other halogenated thiacetazone derivatives were also assayed for their anti-TB activities and the results of compelementary tests are presented in Table 2.

**Table 2:** MICs for the most potent compounds against *M*. *tuberculosis *H_37_R_V, _Erdman and a few drug-resistant strains

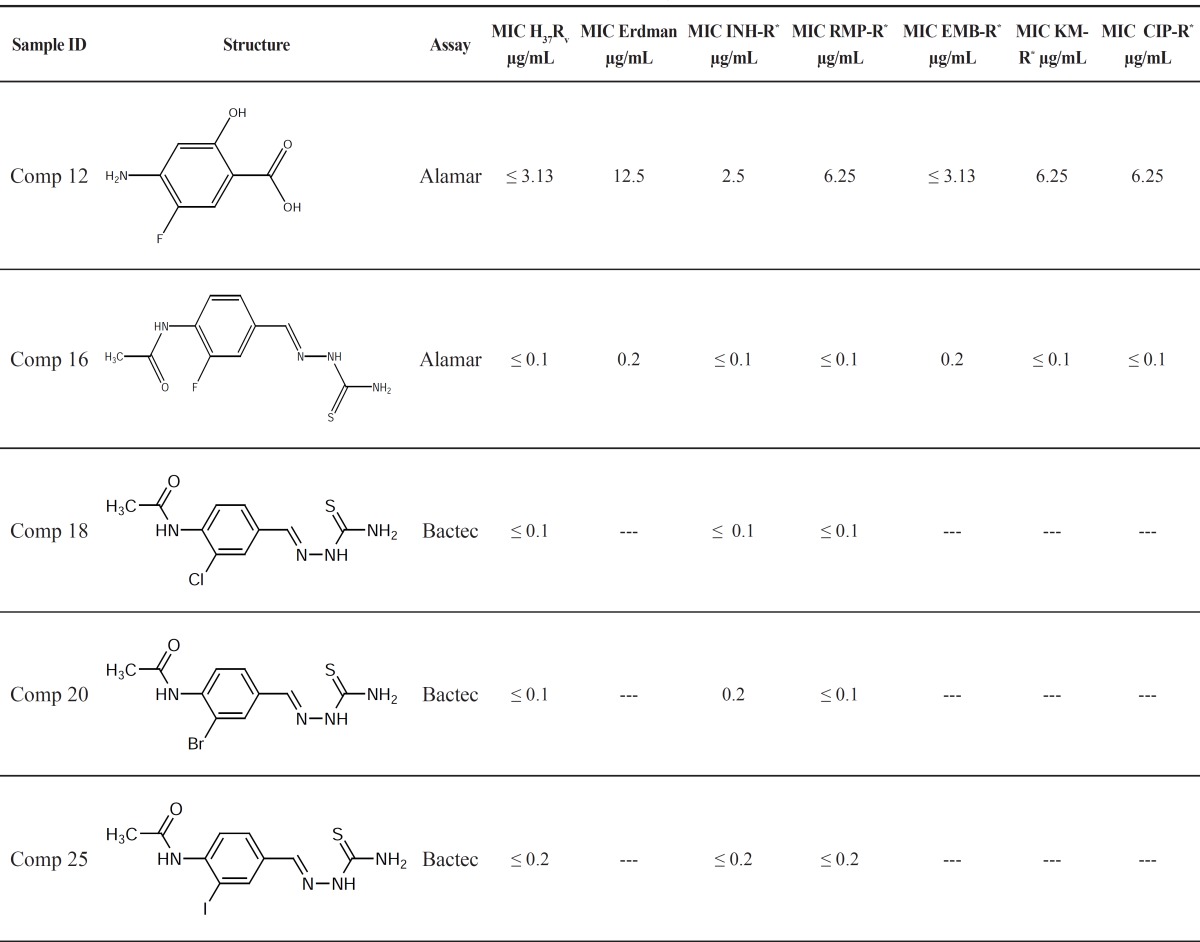

*INH-R:Isoniazid resistant, EMB-R: Ethambutol resistant, RMP-R:Rifampin resistant, KM-R: Kanamycin resistant, CIP-R: Ciprofloxacin resistant.

As it appears from Table 1 and 2, all halogenated derivatives of thiacetazone were more potent than thiacetazone against *M*. *tuberculosis *H_37_R_V_. The higher activities of all halogenated derivatives compared to the parent compound (thiacetazone) could be due to the effect of halogen substituents on electronic and/or partitioning characteristics of thiacetazone.

Comparison of MIC values for the halogenated derivatives (comp 16-25) shows the following order of activity:

F > Cl = Br > I

The decrease in activity when a halogen substituent is changed with its larger counterpart, might be due to the steric hindrance caused by larger halogen substituents on aromatic ring. The minimum bactericidal concentration (MBC) for compounds 12 and 16 against a few M. Tuberculosis strains are presented in Table 3.

**Table 3:** MBCs of compounds 12 and 16 against *M.* *tuberculosis *H_37_R_V _and a few drug-resistant strains.

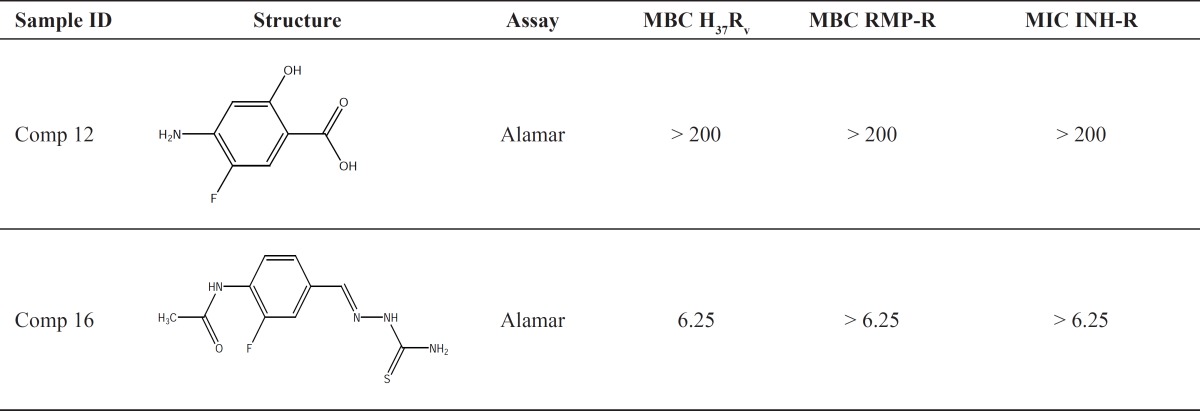

In summary the result of this study shows that substitution of a halogen group on the phenyl ring in thiacetazone, improves anti-*M.* *tuberculosis* activity of this compound. Interestingly the best halogen substituent is fluorine which has the smallest size from one hand and the strongest electronegativity from the other hand among the halogen atoms. This fact reemphasizes the unique nature of fluorine as a golden substituent in medicinal chemistry.

Thiacetazone is a thiocarbamide-containig drug which has been reported to alter the mycolic acid profile of M.bovis BCG (13). Mycolic acids are a complex mixture of branched, long-chain fatty acids, representing key components of the highly lipophilic (hydrophobic) *mycobacterial* cell wall. Pathogenic *mycobacteria* carry mycolic acids that contain cyclopropane rings in their structure. The enzyme which is responsible for cyclopropanation of mycolic acid is cyclopropane mycolic acid synthases (CMASs) and it has been proven that thiacetazone and its analogs act directly on CMASs and suppress the biosynthesis of cyclopropane-containing mycolic acids. Thiosemicarbazone moiety is recognized as the pharmacophore group in thiacetazone scaffold 13-15). Therefore it could be speculated that the new thiacetazone-related compounds which are reported in this study, exert their activity by a similar mechanism. While this speculation is attractive, further confirmation is required through complementary mechanistic studies. *In-vivo* screening will also clarify the effectiveness of these compounds in biological fluids. 

## Conclusion

In this work, we present the synthesis of fluorinated derivatives of three official anti- *M.tuberculosis* drugs: pyrazinamide, PAS and thiacetazone. Contrary to pyrazinamide and PAS, the fluoro substituted thiacetazone exhibited higher activity than thiacetazone. Therefore, this study suggests the incorporation of a fluoro substituent on position 3 of thiacetazone in order to better interaction with its possible target cyclopropane mycolic acid synthase (CMAS). Other halogenated derivatives were also more active than thioacetazone but fluoro derivative was the most potent analog.

In summary, this study demonstrated that simple structural modification of the thioacetazone scaffold may lead to a new thiacetazone analog for the potential treatment of *tuberculosis* as mycolic acid inhibitor.
